# Synthesis and Biological Activity Characterization of Vascular Endothelial Growth Factor Using an Optimized Wheat Germ Cell-Free System

**DOI:** 10.3390/cimb48030290

**Published:** 2026-03-09

**Authors:** Ming Liu, Ran Xiao, Chuiyang Kong, Aimei Liao, Long Pan, Jihong Huang

**Affiliations:** 1 College of Grain and Food Science, Henan University of Technology, Zhengzhou 450001, China; lomine55@163.com (M.L.);; 2 Food Laboratory of Zhongyuan, Henan University of Technology, Zhengzhou 450001, China; 3 College of Agriculture, Henan University, Kaifeng 475004, China; 4 School of Food and Pharmacy, Xuchang University, Xuchang 461000, China

**Keywords:** recombinant protein, cell-free protein synthesis, growth factors, bioactivity

## Abstract

Cell-free protein synthesis has become a powerful tool for producing functional proteins, circumventing many limitations of live-cell systems. Platforms based on wheat germ extract are favored for their high efficiency in translating and folding complex eukaryotic proteins. To overcome the energy limitation common in such systems, we engineered an *Escherichia coli* strain to function as a self-renewing ATP source. This strain co-expresses a three-enzyme cascade—adenosine kinase, adenylate kinase, and acetate kinase—that efficiently converts adenosine and acetyl phosphate into ATP. Using the lysate from this biocatalyst to energize an optimized wheat germ extract, we established a high-performance cell-free synthesis platform. This integrated system supported the robust production of multiple recombinant proteins. As a key demonstration, we synthesized human vascular endothelial growth factor 165, which exhibited full biological activity. The cell-free-produced VEGF_165_ significantly stimulated the proliferation of human umbilical vein endothelial cells (HUVECs) and human skin fibroblasts (HSFs). It also potently induced angiogenic responses, including the formation of extensive, interconnected capillary-like networks by HUVECs in vitro and accelerated cell migration in scratch-wound assays. Our work establishes a scalable and efficient platform for on-demand production of bioactive eukaryotic proteins, highlighting its considerable potential for advancing regenerative medicine and related therapeutic applications.

## 1. Introduction

Cell-free protein synthesis (CFPS) has emerged as a transformative platform that bypasses the constraints of living cells by reconstituting the core transcriptional and translational machinery in an open and controllable environment [1]. This system offers unique advantages for biochemical research and synthetic biology. For example, by eliminating the cell membrane barrier, CFPS enables direct, real-time access and precise manipulation of all components—from DNA templates and ribosomes to energy sources and building blocks [2,3]. Second, protein synthesis is typically achieved within hours, substantially faster than conventional cell-based systems [4]. Additionally, CFPS can directly utilize PCR products as expression templates, circumventing time-consuming steps such as plasmid construction and induction strategies [5]. Most notably, CFPS enables the production of proteins that are cytotoxic or difficult to express in living cells [6]. These advantages have established CFPS as an indispensable tool for rapid prototyping and high-throughput testing of genetic circuits and complex metabolic pathways, substantially accelerating design–build–test cycles in biological engineering [7,8]. Enhancing the efficiency and robustness of CFPS systems is therefore critical to fully realize their potential in biotechnology and biomedical applications. Currently, various CFPS systems have been developed, including those derived from Escherichia coli, insect cells, mammalian cells, and wheat germ. Each system presents distinct advantages and limitations. A summary of the characteristics, commercial availability, and reported protein yields of each CFPS system is provided in Table 1. Among these, the wheat germ extract (WGE)-based CFPS system offers notable advantages for eukaryotic protein expression. Typically, codon optimization is not required for in vitro expression in this system. Moreover, the WGE system enables high-yield production of eukaryotic proteins with enhanced solubility—a distinctive feature among CFPS platforms [9]. Therefore, we selected the WGE system for in vitro translation in this study, aiming to achieve substantial expression levels and high functional activity of the target protein.

Enhancing CFPS productivity requires meticulous optimization of the reaction environment to maximize resource utilization and protein output. This involves fine-tuning fundamental parameters such as pH, which must be adjusted according to the specific extract source—whether bacterial or eukaryotic—to optimize ribosomal activity [10,11]. Temperature is another critical factor, as it must balance reaction kinetics with proper protein folding [12]. The concentrations of essential ions, including Mg^2+^ and K^+^, also require precise optimization, as they are crucial for translation initiation, ribosome function, and tRNA binding [13,14]. However, the most pervasive limitation is the sustainable supply of chemical energy. Adenosine triphosphate (ATP) is the indispensable energy currency driving transcription, translation, and chaperone-assisted folding. Relying on a single, stoichiometric addition of ATP is inefficient and costly, while the accumulation of its hydrolysis product, adenosine diphosphate (ADP), can inhibit the reaction [15,16]. Therefore, integrating a robust ATP regeneration system that continuously recycles ADP back to ATP is paramount. Such a system transcends the stoichiometric limits of ATP, sustains prolonged synthesis, and improves the overall economic viability of CFPS.

To address this central challenge, we developed an integrated strategy that combines metabolic engineering with CFPS. We first constructed a synthetic *Escherichia coli* strain engineered with a heterologous multi-enzyme cascade—comprising adenosine kinase (AK), adenylate kinase (ADK), and acetate kinase (ACK)—designed for efficient ATP regeneration from low-cost precursors [17]. After optimizing the catalytic parameters for ATP production, we harnessed the lysate from this whole-cell biocatalyst as an energized feedstock. This ATP-replenishing lysate was used to drive a subsequently optimized wheat germ extract (WGE)-based CFPS system, chosen for its proficiency in producing correctly folded eukaryotic proteins.

To evaluate protein expression in the optimized system, vascular endothelial growth factor 165 (VEGF_165_), a disulfide-linked homodimeric protein, was selected as a model therapeutic protein. VEGF_165_ is a key signaling molecule that binds to specific receptor tyrosine kinases (VEGFRs) on endothelial cells, promoting their proliferation, migration, and tube formation, thereby mediating essential physiological processes including angiogenesis, wound healing, and tissue repair [18,19]. In conventional WGE-based cell-free systems, dithiothreitol (DTT) is typically added to enhance translation efficiency. However, DTT exhibits reducing activity that can disrupt disulfide bonds in proteins. Supplementing the CFPS system with reagents such as endoplasmic reticulum oxidoreductase 1 alpha (ERO1α), protein disulfide isomerase (PDI), glutathione (GSH/GSSG), and thioredoxin (TrxA) in the absence of DTT effectively addresses this limitation, enabling the production of biologically analogous proteins with intact disulfide bonds [20,21,22,23]. Furthermore, key parameters of the WGE-CFPS system were systematically optimized to achieve a balance among protein yield, synthesis rate, and product quality. Finally, comprehensive cell-based biocompatibility assays were performed to confirm the robust biological activity of the recombinant VEGF165 produced by CFPS. This work establishes a platform for the on-demand production of functional eukaryotic proteins, offering a scalable and economically attractive framework for applications in regenerative medicine and industrial protein synthesis.

## 2. Materials and Methods

### 2.1. Materials and Culture Conditions

All plasmids, strains and primers used in this study are listed in Table 2. The pET28a plasmid was purchased from Sangon Biotech (Shanghai, China), and the pEU plasmid was from Tiangen Biotech (Beijing, China). *E. coli* DH5α was used for recombinant plasmid construction, and *E. coli* BL21(DE3) served as the host for the ATP-producing strain. Human umbilical vein endothelial cells (HUVECs) and human skin fibroblasts (HSFs) were obtained from Newgainbio (Wuxi, China). Bacterial strains were cultured in Luria–Bertani (LB) medium at 37 °C with shaking at 200 rpm. HSFs were routinely maintained in DMEM supplemented with 15% fetal bovine serum (FBS), and HUVECs were cultured in DMEM with 10% FBS. All cell lines were incubated at 37 °C under a humidified atmosphere containing 5% CO_2_. Restriction enzymes were from Takara Bio (Tokyo, Japan). Ribonuclease inhibitors and protease inhibitors were purchased from Promega (Madison, WI, USA). The FastPure Gel DNA Extraction Mini Kit was purchased from Vazyme Biotech (Nanjing, China). Primary and secondary antibodies for detection, including the 6 × His-Tag Monoclonal Antibody and HRP-conjugated Goat Anti-Mouse IgG, were sourced from Proteintech Group (Wuhan, China). The Calcein AM/PI Double Staining Kit was from Elabscience (Wuhan, China). Actin-Tracker Red-Rhodamine, Hoechst 33342, Click-iT EdU-488 Cell Proliferation Assay Kit and recombinant protein PDI were obtained from Beyotime Biotechnology (Shanghai, China). His protein purification magnetic beads were purchased from GenScript (Nanjing, China). All other molecular biology reagents and analytical-grade chemicals were supplied by Sangon Biotech or Macklin Biochemical (Shanghai, China).

### 2.2. Codon Optimization and Gene Synthesis

The nucleotide sequences for all target genes: Adenosine Kinase (AK) NC_001142.9, Adenylate Kinase (ADK) NC_000913.3, Acetate Kinase (ACK) NC_002695.2, Internal Ribosome Entry Site (IRES) GenBank: AB673330.1, Vitreoscilla Hemoglobin (VHb) GenBank: L21670.1, Leghemoglobin (LegH) NC_041011.1, superfolder Green fluorescent protein (sfGFP) GenBank: KM229391.1, Porcine Hemoglobin (PH) GenBank: AK348747.1, Brain-Derived Neurotrophic Factor (BDNF) GenBank: KF693491.1, Red fluorescence protein (mCherry) GenBank: PQ677678.1, and Vascular Endothelial Growth Factor 165 (VEGF_165_) GenBank: BT019606.1-were retrieved from the NCBI database (https://www.ncbi.nlm.nih.gov/) on 16 October 2025. To enhance expression in *E. coli*, the coding sequences for AK, ADK, and ACK were codon-optimized using the NovoPro online tool (https://www.novopro.cn/tools/codon-optimization.html) on 16 October 2025. The optimized genes were synthesized de novo by Shanghai Sangon Biotech and subsequently cloned into the pET28a vector. For PCR amplification, 50 µL reactions were prepared containing 2× Phanta Flash Master Mix (Vazyme, Nanjing, China), 200 nM of each forward and reverse primer, and 500 ng of gene template. The thermal cycling protocol was as follows: initial denaturation at 95 °C for 3 min; 30 cycles of denaturation at 95 °C for 15 s, annealing at 60 °C for 15 s, and extension at 72 °C for 1 min; followed by a final extension at 72 °C for 5 min. The amplification products were separated by 1% agarose gel electrophoresis, and the target DNA fragments were purified using a commercial gel extraction kit (Vazyme, Nanjing, China).

### 2.3. Assembly of Tandem AK, ADK, and ACK Gene Constructs

Tandem gene constructs were assembled based on polycistronic design principles. For the primary polycistronic assembly (pET28a-aadck), the genes for AK, ADK, and ACK were PCR-amplified using primers (Table 2) engineered with homologous overhangs of appropriate length for subsequent fusion. A sequence encoding a 6 × His tag was incorporated at the C-terminus of each gene. An alternative construct utilizing internal ribosome entry site (IRES) and 2A peptide elements was also generated (pET28a-AADCK). The assembly was performed via a multi-stage overlap extension PCR protocol [24]. The final amplified product was separated by 1% agarose gel electrophoresis, and the correct DNA fragment was excised and purified using a commercial gel extraction kit.

### 2.4. Construction of Recombinant Expression Plasmids

Purified PCR products containing the target genes and the pET28a/pEU destination vectors were digested with *XhoI* and *BamHI* restriction enzymes. The resulting gene fragments were ligated into the linearized vector backbones using T4 DNA ligase. The ligation products were transformed into chemically competent *E. coli* DH5α cells via heat shock and plated on LB agar containing the appropriate antibiotic for selection. Putative positive clones were initially identified by colony PCR. Selected colonies were cultured in liquid medium for plasmid propagation. The recombinant plasmids were subsequently extracted, verified by restriction analysis, and stored at –20 °C for further use.

### 2.5. Recombinant Protein Expression

For recombinant protein expression, a single colony of the engineered *E. coli* strain was inoculated into 10 mL of LB medium supplemented with kanamycin and grown to mid-log phase (OD_600_ of 0.6–0.8). Protein production was induced by adding 0.1 mM isopropyl β-D-1-thiogalactopyranoside (IPTG), followed by incubation at 16 °C overnight. Cells were harvested by centrifugation at 10,000× *g* for 10 min at 4 °C. The pellet was resuspended and subjected to ultrasonic disruption at 450 W. The resulting cell lysate was separated by centrifugation into soluble (supernatant) and insoluble (sediment) fractions. Protein expression levels and solubility were subsequently analyzed by sodium dodecyl sulfate–polyacrylamide gel electrophoresis (SDS-PAGE) and Western blotting.

### 2.6. Whole-Cell Catalysis for ATP Production

Following induction, cells were harvested by centrifugation at 12,000× *g* for 5 min at 4 °C. The resulting pellet was weighed and resuspended in an appropriate volume of borate buffer. Whole-cell biocatalytic reactions were performed in 1.5 mL Eppendorf tubes containing defined concentrations of adenosine, Mg^2+^, lithium acetyl phosphate (ACP), and cells resuspended in PBS. The reaction mixture was thoroughly mixed by vortexing and incubated at 35 °C. The concentration of each component was systematically optimized to maximize ATP yield. Subsequently, samples were centrifuged at 12,000 rpm for 10 min at 4 °C. The supernatant was collected and filtered through a 0.22 μm aqueous membrane. The filtrate was appropriately diluted, and ATP production was quantified by high-performance liquid chromatography (HPLC).

### 2.7. Preparation of Wheat Germ Extract (WGE)

Wheat germ extract was prepared following a protocol adapted from [25]. Briefly, intact wheat germs were selected and repeatedly washed. The washed germs were then ground in a pre-chilled mortar with an equal volume of extraction buffer (80 mM HEPES-KOH, pH 7.6, 200 mM potassium acetate, 10 mM magnesium acetate, 4 mM CaCl_2_) to form a homogeneous paste. The mixture was subjected to two rounds of ultracentrifugation at 30,000× *g* for 30 min at 4 °C. The clarified supernatant, collected from between the lipid layer and pellet, was further purified by gel filtration through Sephadex G-25. The final extract was aliquoted, flash-frozen in liquid nitrogen, and stored at –80 °C.

### 2.8. In Vitro Transcription (IVT)

IVT was performed according to a previously described method with minor modifications [26]. Briefly, a 50 µL reaction mixture was assembled containing 1× transcription buffer (final concentration: 16 mM HEPES-KOH, pH 7.8, 3.2 mM magnesium acetate, 0.4 mM spermidine, 2 mM DTT), 2.5 mM of each NTP, 1 µg of PCR-amplified DNA template, 40 U of Sp6 RNA polymerase, and 40 U of RNase inhibitor. The reaction was incubated at 37 °C in a heat block for 3 to 6 h. Following incubation, the synthesized mRNA concentration was quantified using a NanoDrop spectrophotometer (Thermo Fisher Scientific, Waltham, MA, USA), and its integrity was assessed by separation on a 1% agarose gel.

### 2.9. Protein Expression and Detection

A bilayer system for cell-free translation was established based on a previously described method with slight modifications [27,28]. Briefly, the whole-cell catalysis reaction mixture obtained from the previous step was lysed and centrifuged at 30,000× *g* for 30 min at 4 °C. After appropriate dilution, the supernatant was used as the translation supplement in the upper layer. The upper reaction mixture (100 µL) contained 30 mM HEPES-KOH (pH 7.8), 100 mM potassium acetate, 2.7 mM magnesium acetate, 0.4 mM spermine, 0.3 mM amino acid mixture, 0.25 mM GTP, 16 mM creatine phosphate, and 1× protease inhibitor cocktail. The lower layer (20 µL) consisted of 200 µg mRNA, 9 µL wheat germ extract, 40 U RNase inhibitor, and 0.5 µL 10 mg/mL creatine kinase. To assess the functional expression of proteins containing disulfide bonds, DTT was omitted from the translation supplement, and 0.2 mg/mL PDI was added to the lower translation mixture. The assembled reactions were incubated in a thermocycler at 15 °C for 15 h. Protein synthesis was confirmed by laser scanning confocal microscopy (LSM 900, Carl Zeiss, Oberkochen, Germany), and relative expression levels were evaluated by Western blotting.

### 2.10. VEGF_165_ Purification

After completion of the WGE reaction, the mixture was centrifuged at 12,000× *g* for 10 min at 4 °C to remove insoluble precipitates. The target protein was then purified using Ni-charged MagBeads (Genscript, Piscataway, NJ, USA) strictly following the manufacturer’s instructions. The resulting VEGF-containing protein solution was subsequently subjected to ultrafiltration for desalting using centrifugal filter units with a 3 kDa molecular weight cutoff (Merck, Darmstadt, Germany) and concentrated by centrifugation at 4000× *g* and 4 °C to thoroughly remove small-molecule impurities. Finally, the purified protein was diluted in PBS and quantified using an ELISA kit (H2158, Elabscience, Wuhan) according to the manufacturer’s protocol. The resulting VEGF_165_ solution was diluted to working concentrations for subsequent cell-based assays.

### 2.11. Assessment of Cell Proliferation and Viability

#### 2.11.1. Cell Viability Assay

The bioactivity of cell-free synthesized VEGF_165_ was evaluated using a CCK-8 assay kit (Solarbio, Beijing, China). HUVEC and HSF cells were seeded in 96-well plates at a density of 6 × 10^3^ cells per well and cultured for 24 h under standard conditions (37 °C, 5% CO_2_). Cells were then treated with the recombinant VEGF_165_ for 1 day, with medium-only treated cells serving as the negative control group. After treatment, the supernatant was removed, cells were washed twice with PBS, and the CCK-8 reagent was added according to the manufacturer’s instructions. Following a 4 h incubation, the absorbance of each well was measured at 450 nm using a microplate reader (Leica, Wetzlar, Germany). All experiments were performed in triplicate. Cell viability was calculated using the following formula:

(1) $$\mathrm{Cell}\;\mathrm{viability}\;\%\;=\frac{A_{1}-A_{0}}{A_{2}-A_{0}}\;\times \;100\%$$

where A_0_ is the absorbance of wells containing medium and CCK-8 reagent without cells, A_1_ is the absorbance of wells with cells, CCK-8, and VEGF_165_, and A_2_ is the absorbance of wells with cells and CCK-8 reagent only (without VEGF_165_).

#### 2.11.2. Cell Morphological Observation

To assess the impact of recombinant purified or not purified VEGF_165_ on cellular morphology and cytoskeletal architecture, we performed fluorescence staining of cytoskeleton and nuclei followed by confocal microscopy. HUVEC and HSF cells were seeded in 24-well plates at a density of 2 × 10^4^ cells per well and allowed to adhere for 12 h. Following attachment, cells were either left untreated (negative control) or treated with recombinant VEGF_165_ for an additional 12 h. Subsequently, cells were fixed and sequentially stained with Actin-Tracker Red-Rhodamine to visualize F-actin and Hoechst 33342 to label nuclei. Images were acquired using LSM 900.

#### 2.11.3. Cell Morphological Observation

To evaluate the cytotoxic effects of purified and unpurified VEGF_165_, HUVEC and HSF cells were seeded uniformly into 24-well plates at a density of 2 × 10^4^ cells per well and cultured under standard conditions (37 °C, 5% CO_2_, saturated humidity) for 24 h to allow full adhesion. After discarding the original medium, the cells were incubated with 1 mL of fresh medium containing appropriate concentrations of purified or unpurified VEGF_165_. Following 24 h of treatment, cell viability was assessed using a Calcein AM/PI double staining kit according to the manufacturer’s instructions. Stained cells were visualized and imaged by fluorescence microscopy, with live cells exhibiting green fluorescence (Calcein AM) and dead cells showing red fluorescence (PI), enabling evaluation of cell survival and death across treatment groups.

#### 2.11.4. Cell Proliferation Assay

Cell proliferation was evaluated using a 5-ethynyl-2′-deoxyuridine (EdU) incorporation assay. Cells were seeded in 24-well plates at a density of 2 × 10^4^ cells per well and cultured for 24 h under standard conditions (37 °C, 5% CO_2_). After attachment, cells were treated with the recombinant VEGF_165_ protein. An equal volume of 2× EdU working solution was then added to each well, followed by a 3 h incubation. The medium was subsequently removed, and cells were washed twice with cold PBS, fixed with 4% paraformaldehyde for 15 min at room temperature, and permeabilized with 0.03% Triton X-100 for 10 min. The incorporated EdU was labeled by incubating the cells with 500 µL of 1× EdU click reaction mixture for 30 min in the dark. Finally, cell nuclei were counterstained with Hoechst 33342. Fluorescent images were acquired using a fluorescence microscope (LSM 900, Carl Zeiss, Oberkochen, Germany) to assess the pro-proliferative effect of VEGF_165_.

#### 2.11.5. Scratch Wound Healing Assay

For the scratch test, HUVECs and HSFs were plated in a 6-well plate and grown to 90% confluency. The cell monolayer was scratched with a 200 μL pipette tip, and then washed and supplemented treated with PBS and Recombinant VEGF_165_ (150 pg/mL). Images were taken at 0, 6, 12 and 24 h using a microscope. The percentage of cell migration was calculated using the following formula:

(2) $$\mathrm{Migration}\;\mathrm{rate}\;\%\;=\frac{S_{1}-S_{0}}{S_{0}}\;\times \;100\%$$

where S_0_ is the initial scratch area at 0 h and S_1_ is the scratch area at different time.

#### 2.11.6. Cell Migration Assay

For the Transwell assay, cells were seeded in the upper chamber, while the lower chamber contained culture medium with different treatments (PBS and Recombinant VEGF_165_ (150 pg/mL)). After 36 h of incubation, the membrane of the upper chamber was carefully removed with a cotton swab, and the cells in the lower chamber were fixed in 4% paraformaldehyde for 30 min at room temperature and then stained with crystal violet and observed under an inverted microscope.

#### 2.11.7. Tube Formation Assay

The pro-angiogenic effect of recombinant purified or not purified VEGF_165_ was evaluated using a Matrigel-based in vitro tube formation assay. Matrigel solution was thawed overnight at 4 °C, aliquoted into a 24-well plate, and allowed to polymerize at 37 °C for 1 h to form a solid gel. HUVECs were then seeded onto the polymerized Matrigel at a density of 2 × 10^5^ cells per well in medium supplemented with VEGF_165_ (150 pg/mL). After a 4 h incubation at 37 °C under 5% CO_2_, the medium was removed, and live cells were stained by incubating with 2 µM Calcein-AM working solution for 30 min at 37 °C in the dark. Fluorescent images of the tubular networks were acquired using a fluorescence microscope. Quantitative analysis of total tube length and branch points was performed using ImageJ software ((1.54p version)) with an angiogenesis analysis plugin to objectively assess the regulatory role of VEGF_165_ in angiogenesis.

### 2.12. Statistical Analysis

All data are expressed as the mean ± standard deviation (SD). Independent-sample *t*-tests were used for comparisons between among groups. One-way analysis of variance (ANOVA) using SPSS software (26.0 version) determined significance, with *p* < 0.05 considered statistically significant. Graphs were generated with OriginLab, where * *p* < 0.05, ** *p* < 0.01, and *** *p* < 0.001.

## 3. Results and Discussion

### 3.1. Construct the pET28-aadck and pET28-AADCK Expression Vectors

**Figure 1:**
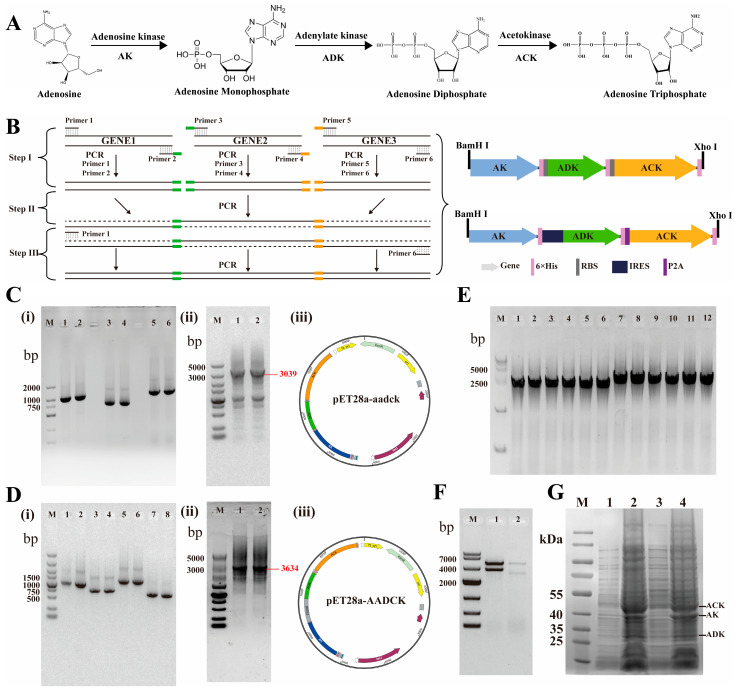
Construction and validation of the recombinant expression vectors pET28-aadck and pET28-AADCK for an ATP-regenerating enzyme cascade. (**A**) Schematic of the ATP synthesis pathway illustrating the substrate conversions catalyzed by AK, ADK and ACK. (**B**) Strategy for assembling tandem open reading frames via overlap extension PCR. (**C**) Assembly of the pET28-aadck construct: (**i**) Agarose gel analysis of PCR-amplified gene fragments (AK, ADK, ACK) and the IRES element, each engineered with homologous overlapping ends; (**ii**) Product of the overlap extension PCR assembly; (**iii**) Map of the final recombinant plasmid pET28-aadck. (**D**) Assembly of the pET28-AADCK construct: (**i**) PCR-amplified AK, ADK, and ACK fragments with homologous ends; (**ii**) Corresponding overlap extension PCR product; (**iii**) Map of the final recombinant plasmid pET28-AADCK. (**E**) Colony PCR screening confirms the successful cloning of both plasmids (lanes 1–6: pET28-aadck; lanes 7–12: pET28-AADCK). (**F**) Analytical restriction digestion of the recombinant plasmids: Lane M, 10,000 bp DNA ladder; lane 1, pET28-AADCK digested with XhoI and BamHI; lane 2, pET28-aadck digested with XhoI and BamHI. (**G**) SDS-PAGE analysis confirms protein expression following IPTG induction in *E. coli*: lanes 1–2, soluble lysate and insoluble pellet from cells harboring pET28-AADCK; lanes 3–4, soluble lysate and insoluble pellet from cells harboring pET28-aadck.

Regenerating ATP is a critical and often limiting factor in biomanufacturing processes that rely on ATP-dependent enzymes. To address this, we engineered an efficient, heterologous ATP regeneration system in Escherichia coli based on a three-enzyme cascade. This system comprises AK, ADK, and ACK, which work sequentially to convert adenosine into ATP [15]. AK catalyzes the phosphorylation of adenosine to adenosine monophosphate (AMP), ADK mediates the conversion of AMP to ADP, and ACK completes the cycle by phosphorylating ADP to ATP using acetyl phosphate, thereby effectively managing intracellular energy balance (Figure 1A). We report the successful construction, expression, and functional validation of this synthetic pathway, demonstrating its potential as a versatile platform for ATP-driven biosynthesis in a whole-cell biocatalyst. To construct this pathway, the genes encoding AK, ADK, and ACK were assembled into tandem expression cassettes. Using an overlap extension PCR strategy, each gene was first amplified with primers (Table 2) designed to append homologous overlapping sequences, enabling their subsequent fusion in a single, one-pot reaction (Figure 1B). The individual PCR products for AK, ADK, ACK, and an IRES element were verified by agarose gel electrophoresis (1% *w*/*v*), showing bands consistent with their expected sizes (Figure 1C(i),D(i)). These fragments were purified, assembled via overlap extension PCR, and the final tandem constructs were analyzed by electrophoresis. The assembly product for the ak-adk-ack cassette showed a predominant, clear band at approximately 3000 bp, matching the theoretical size of 3039 bp for the fragment designed for insertion between the BamHI and XhoI sites in plasmid pET-aadck (Figure 1C(ii)). A corresponding larger band (>3000 bp) was observed for the construct containing the additional IRES element (pET-AADCK, theoretical size 3634 bp; Figure 1D(ii)). Following gel extraction and ligation, colony PCR and double restriction enzyme digestion confirmed the successful cloning of both recombinant plasmids, pET-aadck and pET-AADCK, in *E. coli* DH5α (Figure 1E,F). The recombinant plasmids were transformed into *E. coli* BL21(DE3) for protein expression. Induction with IPTG led to the co-expression of the three kinases. SDS-PAGE analysis of the total cell lysate revealed distinct protein bands corresponding to the theoretical molecular weights of AK (~40 kDa, observed near the 40 kDa marker), ADK (~27 kDa, observed above the 25 kDa marker), and ACK (~44 kDa, observed above the 40 kDa marker) (Figure 1G). The observed results suggest successful heterologous expression of the three enzymes; however, quantification of their expression levels requires confirmation by Western blot analysis, as each enzyme carries a C-terminal 6 × His tag.

### 3.2. Expression of Gene AK, ADK and ACK in E. coli

**Figure 2:**
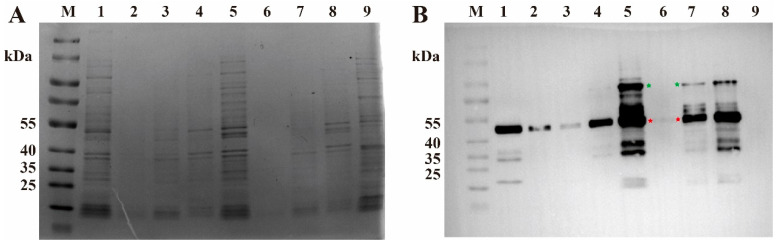
Analysis of heterologous protein expression by SDS-PAGE and Western blot. (**A**) SDS-PAGE analysis of cell lysates. Lane M, protein molecular weight marker (10–180 kDa). Lanes 1–4: *E. coli* expressing the pET28-aadck plasmid induced at 16 °C (lane 1, whole cell lysate; lane 2, supernatant) or 25 °C (lane 3, supernatant; lane 4, whole cell lysate). Lanes 5–8: *E. coli* expressing the pET28-AADCK plasmid induced at 16 °C (lane 5, whole cell lysate; lane 6, supernatant) or 25 °C (lane 7, supernatant; lane 8, whole cell lysate). Lane 9: *E. coli* BL21 (DE3) containing the empty pET28a vector (negative control). (**B**) Corresponding Western blot probed with an anti-His tag antibody. (The red module represents ak, and the green module represents adk-ack).

To assess the correct expression of each enzyme, cells harboring both plasmids were induced at different temperatures, diluted, and subsequently analyzed by SDS-PAGE and Western blot. Given the cascade order of AK–ADK–ACK, adenylate kinase was expected to exhibit the highest expression level, as reflected by the prominent band near 40 kDa in Figure 2B. However, in the strain carrying pET28-aadck, AK was expressed normally, whereas ADK and ACK showed negligible expression regardless of the induction temperature. Analysis of the sequencing data revealed that the ribosome-binding site (RBS) was positioned unusually close to the start codon of these genes, which likely contributed to their low or undetectable expression. Previous studies indicate that an optimal increase in the distance between the Shine–Dalgarno sequence and the start codon can enhance translational efficiency, although excessive spacing may be detrimental [29]. Moreover, single-nucleotide variations within the RBS can substantially alter translational strength, thereby enabling a broad range of expression levels [30]. In contrast, strong expression of AK across different temperatures was observed in the strain carrying pET28-AADCK (Figure 2B), confirming the correct construction of the prokaryotic expression vector. We further investigated whether the IRES and the 2A peptide could function in the prokaryotic system. The EMCV-derived IRES indeed mediated translation in prokaryotes, as evidenced by additional protein bands beyond the AK signal [31]. However, to our knowledge, when linked by a P2A sequence, the two proteins are primarily expressed as a fusion protein in prokaryotic systems while retaining biological activity [32]. Therefore, our results demonstrate successful expression of all three proteins involved in the ATP synthesis pathway in constructs designed with IRES and P2A elements, although ADK and ACK were produced as a fusion protein.

### 3.3. Whole-Cell Catalytic Production of ATP

**Figure 3:**
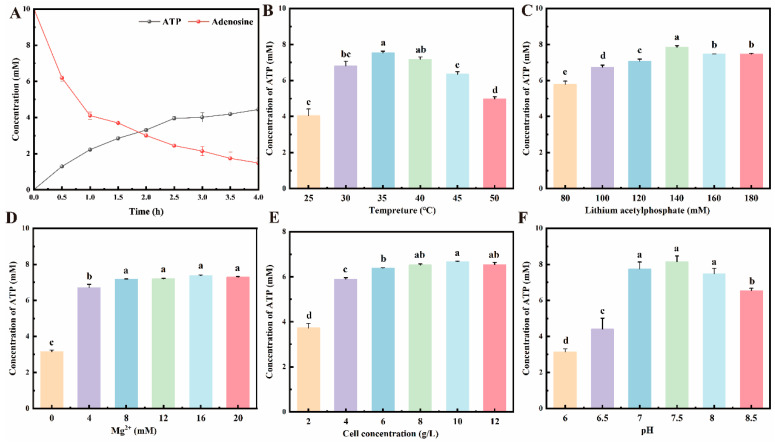
Systematic optimization of reaction parameters for whole-cell ATP synthesis. The synthesis efficiency was evaluated as a function of six key variables: (**A**) reaction time, (**B**) temperature, (**C**) ACP concentration, (**D**) Mg^2+^ concentration, (**E**) whole-cell catalyst (bacterial) concentration, and (**F**) pH. Data are presented as mean ± SD; statistical significance between groups is denoted by lowercase letters at *p* < 0.05 (*n* = 3).

Through systematic optimization of a heterologous three-enzyme cascade for ATP regeneration, we delineated the effects of key reaction parameters—time, temperature, ACP and Mg^2+^ concentrations, cell density, and pH—with results consolidated in Figure 3. ATP accumulation followed classical Michaelis-Menten kinetics over a 4.5 h period [33], exhibiting rapid linear synthesis (~3.5 mM within 2 h) before plateauing near 4.0 mM, a transition indicative of shifting limitations from substrate availability to potential product inhibition or reduced enzyme stability, establishing an optimal reaction window of 3.5 h. A pronounced bell-shaped dependence on temperature was observed, with a clear optimum at 35 °C yielding approximately 7.5 mM ATP; lower temperatures (<30 °C) likely constrained enzymatic activity and membrane fluidity, whereas higher temperatures (>40 °C) precipitated a sharp decline in yield, attributable to partial protein denaturation and loss of essential cofactors [34,35]. The concentration of the key substrate ACP demonstrated a critical nonlinear relationship: while increasing from 80 mM to 140 mM boosted ATP yield from 5.0 mM to 8.0 mM, further escalation to 180 mM suppressed output to ~7.0 mM, suggesting significant substrate inhibition or induction of cellular osmotic stress [36]. As an essential catalytic cofactor, Mg^2+^ concentration was positively correlated with productivity up to 16 mM, enhancing yield to approximately 7 mM by stabilizing enzyme architecture and facilitating phosphate transfer, beyond which no further benefit was observed and risks of ionic toxicity increased. Varying the concentration of the whole-cell biocatalyst revealed a threshold effect; ATP yield increased substantially from 2 mM to 6.5 mM as cell density was raised from 2 to 8 g/L (cell dry weight), but further increases to 12 g/L provided no additional gain, likely due to emerging limitations in oxygen transfer, substrate diffusion, and metabolite accumulation [2]. Finally, the reaction exhibited a sharp pH optimum at 7.5, yielding about 8 mM ATP, with activity precipitously declining on either side of this narrow range, underscoring the precise requirement for maintaining the protonation state of active-site residues and the binding competence of Mg^2+^ [37]. Collectively, this multi-parameter optimization defines a refined operational framework—3.5 h, 35 °C, 140 mM ACP, 16 mM Mg^2+^, 8 g/L cells, pH 7.5—that maximizes the ATP synthesis capability of the engineered system for subsequent biocatalytic applications.

### 3.4. Construction of Cell-Free Expression System

**Figure 4:**
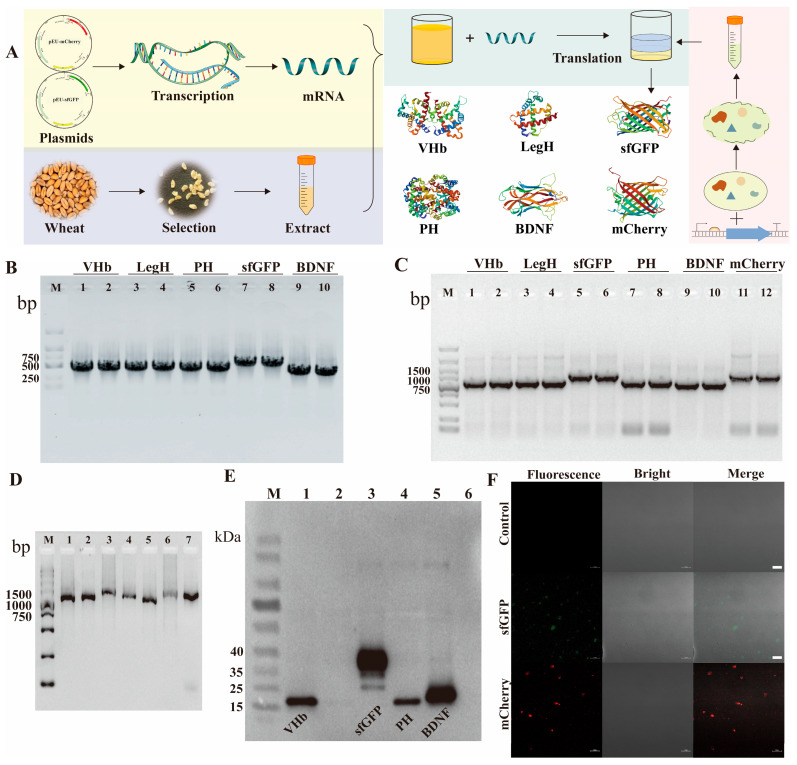
Results of the Cell-Free System Construction. (**A**) A schematic overview of the workflow, beginning with DNA template preparation, followed by IVT to mRNA, incubation with the wheat germ extract (WGE) reaction mixture, and final protein analysis. (**B**) Agarose gel electrophoresis of PCR-amplified gene fragments: lanes 1–2, VHb; 3–4, LegH; 5–6, PH; 7–8, sfGFP; 9–10, BDNF. (**C**) Analysis of the corresponding linearized DNA transcription templates for each gene: lanes 1–2, VHb; 3–4, LegH; 5–6, sfGFP; 7–8, PH; 9–10, BDNF; 11–12, mCherry. (**D**) IVT results showing synthesized mRNA for each target (lanes 1–6: VHb, LegH, sfGFP, PH, BDNF, mCherry) and a DNA control (lane 7). (**E**) SDS-PAGE confirming the in vitro translation of selected proteins: VHb (lane 1), LegH (lane 2), sfGFP (lane 3), PH (lane 4), and BDNF (lane 5). (**F**) Laser scanning confocal microscopy images verifying the fluorescence and proper folding of expressed sfGFP and mCherry; scale bar, 10 µm.

A robust eukaryotic CFPS platform based on WGE was established, successfully expressing six recombinant proteins: VHb, LegH, PH, sfGFP, BDNF, and mCherry. The integrated workflow encompassed DNA template construction, IVT, and translation within the WGE reaction system (Figure 4A). High-fidelity DNA templates are essential for reliable CFPS. Agarose gel electrophoresis confirmed the integrity of PCR-amplified target gene fragments, each showing a single band corresponding to its expected length (Figure 4B). These fragments were subsequently used to generate linear DNA transcription templates containing an SP6 promoter, the target gene, and a poly-A tail. Purified templates, verified by electrophoresis (Figure 4C), served as substrates for IVT. Successful mRNA synthesis was confirmed (Figure 4D), with yields exceeding 10 µg per reaction as quantified by NanoDrop. Subsequent cell-free translation using these mRNAs produced all target proteins, as detected by Western blot analysis leveraging C-terminal 6 × His tags (Figure 4E). The observed molecular weights aligned with theoretical predictions: VHb, LegH, PH, and BDNF migrated near 20 kDa, while sfGFP was detected at approximately 35 kDa. This indicates effective transcription-translation coupling with minimal degradation, though band intensity variations suggest potential influences from codon usage or mRNA secondary structure that warrant future optimization [2]. Finally, direct functional validation was achieved by LSM 900, which confirmed the successful expression and proper folding of the fluorescent proteins sfGFP and mCherry, as evidenced by their respective fluorescence signals (Figure 4F). No signal was detected in negative controls where mRNA was omitted, confirming the specificity of the observed fluorescence. These results collectively validate the capability of our optimized WGE-based CFPS platform to synthesize diverse, functional proteins.

### 3.5. Optimization of Cell-Free Expression System

**Figure 5:**
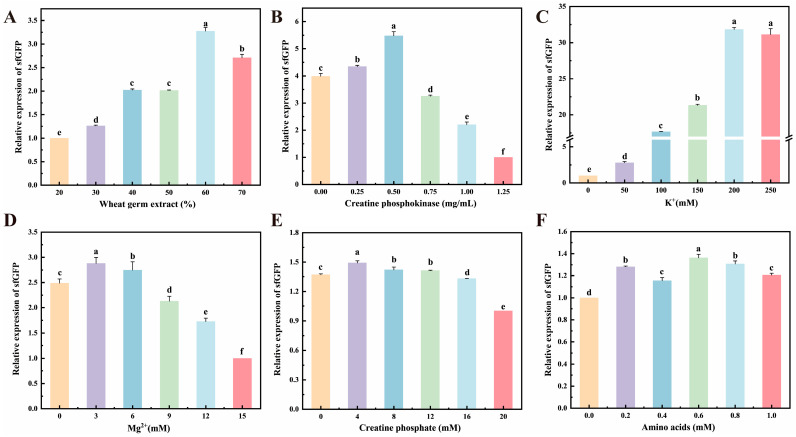
Systematic optimization of a wheat germ-based cell-free protein synthesis system for enhanced sfGFP expression. (**A**) Protein yield as a function of wheat germ extract concentration. (**B**) Effect of creatine kinase concentration on expression efficiency. (**C**,**D**) Optimization of key ionic components: potassium (**C**) and magnesium (**D**) ion concentrations. (**E**) Effect of creatine phosphate concentration on expression efficiency. (**F**) Determination of the optimal amino acid concentration in the reaction mixture. statistical significance between groups is denoted by lowercase letters at *p* < 0.05 (*n* = 3).

The multi-parameter optimization of the CFPS platform based on WGE was conducted to maximize the production of sfGFP. For this purpose, the effects of factors such as WGE concentration, creatine kinase (CrK), potassium ions (K^+^), magnesium ions (Mg^2+^), creatine phosphate (Cr), and amino acid levels were evaluated (Figure 5). The concentration of WGE, a source of ribosomes and translational factors, significantly influenced output; yield increased up to 60% (*v*/*v*) WGE but declined slightly at higher concentrations, potentially due to inhibitory compounds or osmotic imbalance [38] (Figure 5A). CrK, essential for ATP regeneration via the phosphocreatine system, boosted sfGFP synthesis optimally at 0.5 mg/mL, beyond which yields decreased, likely reflecting substrate depletion or feedback inhibition [39] (Figure 5B). Potassium ions (K^+^) were critical for ribosomal integrity and translational initiation, with maximal expression achieved at 200 mM (Figure 5C). Magnesium ions (Mg^2+^) exhibited a bell-shaped response, with an optimum at 3 mM for stabilizing mRNA and facilitating ribosomal subunit assembly; higher concentrations were detrimental, possibly due to non-productive complex formation [14,40] (Figure 5D). Interestingly, Cr concentration was inversely correlated with sfGFP yield in the range of 0–20 mM (Figure 5E). This may be attributed to negative feedback regulation within the ATP-regeneration cycle [41], as the reactions were supplied with ATP from a heterologously expressed kinase cascade. Finally, amino acid concentration showed a threshold-dependent effect: yield increased up to 0.6 mM, plateaued, and declined slightly at higher concentrations, suggesting that translational machinery capacity, rather than substrate availability, becomes limiting [42] (Figure 5F). Collectively, these optimizations defined a refined reaction condition (60% WGE, 0.5 mg/mL CrK, 200 mM K^+^, 3 mM Mg^2+^, 0.6 mM amino acids, 4 mM creatine phosphate) that establishes an efficient eukaryotic CFPS platform.

### 3.6. Biological Activity of VEGF_165_ Produced by CFPS

**Figure 6:**
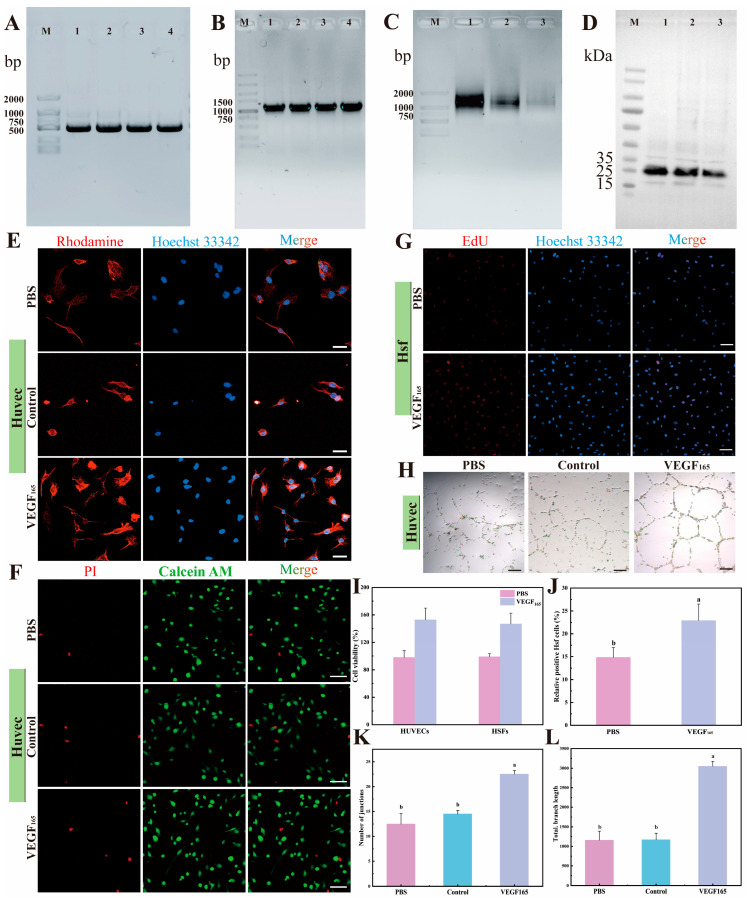
In vitro expression of recombinant VEGF_165_ and its functional validation in promoting proliferation and tube formation. (**A**) Cloning of the VEGF_165_ gene. (**B**) Construction of the template for VEGF_165_ IVT. (**C**) VEGF_165_ IVT at different time points: lane 1, 6 h; lane 2, 3 h; lane 1, 1 h. (**D**) Western blot analysis following in vitro expression of recombinant VEGF_165_. (**E**) Representative images depicting morphological changes in HUVECs treated with recombinant VEGF_165_, scale bar, 40 µm. (**F**) Representative images of live and dead cells of HUVECs treated with recombinant VEGF_165_, scale bar, 100 µm. (**G**) Representative images showing the pro-proliferative effect of recombinant VEGF_165_ on HSF cells, scale bar, 100 µm. (**H**) Representative images of tube formation by HUVECs treated with recombinant VEGF_165_, scale bar, 100 µm. (**I**) Quantitative assessment of viability following treatment of HUVEC and HSF cells with PBS and recombinant VEGF_165_. (**J**) Quantitative analysis of HSF cell proliferation. (**K**,**L**) Quantitative assessment of key parameters for in vitro tube formation by HUVECs treated with recombinant VEGF_165_. Data are presented as mean ± SD; statistical significance between groups is denoted by lowercase letters at *p* < 0.05 (*n* = 3).

To verify the bioactivity of proteins expressed in our cell-free system, VEGF_165_ was selected as a model for a series of biocompatibility studies. The unique identity of the VEGF gene was first confirmed by PCR amplification (Figure 6A), followed by the generation of a linear DNA template optimized for IVT (Figure 6B). IVT time-course experiments yielded approximately 20 µg of mRNA after a 6 h reaction (Figure 6C). Western blot analysis of the cell-free expressed product confirmed successful synthesis, showing a distinct band at the expected molecular weight of ~25 kDa (Figure 6D). The biological activity of the recombinant protein was subsequently evaluated in cell-based assays. Microscopic observation revealed that neither the purified nor the unpurified recombinant VEGF_165_ adversely affected the morphology of HUVECs; cells retained their typical spread morphology with intact cell–cell junctions, indicating preserved structural integrity (Figure 6E). However, differences in cell number were observed: the unpurified VEGF_165_ preparation appeared to contain components that inhibit cell proliferation—such as acetate or trace amounts of *E. coli* lysate, which may be detrimental to cell growth. Cytotoxicity of the recombinant protein was then assessed (Figure 6F,I). The unpurified VEGF_165_ extract diluted in PBS showed no significant difference in cell viability compared to the PBS control, suggesting that trace interfering components in the mixture exhibited minimal cytotoxicity. In contrast, addition of purified VEGF_165_ to HUVEC cultures resulted in a marked increase in cell number, indicating that VEGF_165_ was biologically active and effectively promoted HUVEC proliferation. No significant change was observed in HSF numbers, further confirming the absence of cytotoxicity. Moreover, EdU incorporation assays demonstrated that VEGF_165_ significantly promoted cell entry into DNA synthesis phase, accelerated cell cycle progression, and increased cell proliferation (Figure 6G,I). This proliferative effect is likely mediated through activation of downstream signaling pathways such as MAPK and PI3K/Akt [43]. Finally, tube formation assays revealed robust pro-angiogenic activity of VEGF_165_. Cells in the PBS control group formed only sparse and fragmented tubular structures, and the unpurified VEGF_165_ group showed no significant difference from the PBS control. In contrast, treatment with purified VEGF_165_ induced the formation of dense, interconnected, and complex capillary-like networks (Figure 6H). Quantitative analysis confirmed that VEGF_165_ significantly increased total tube length and the number of branching points (Figure 6J,K), an effect that may be associated with upregulation of key adhesion molecules such as VE-cadherin, which stabilizes endothelial cell junctions [44]. Taken together, these results validate the functional activity of cell-free synthesized VEGF_165_.

### 3.7. Pro-Migratory Effects of VEGF_165_ Produced by CFPS

**Figure 7:**
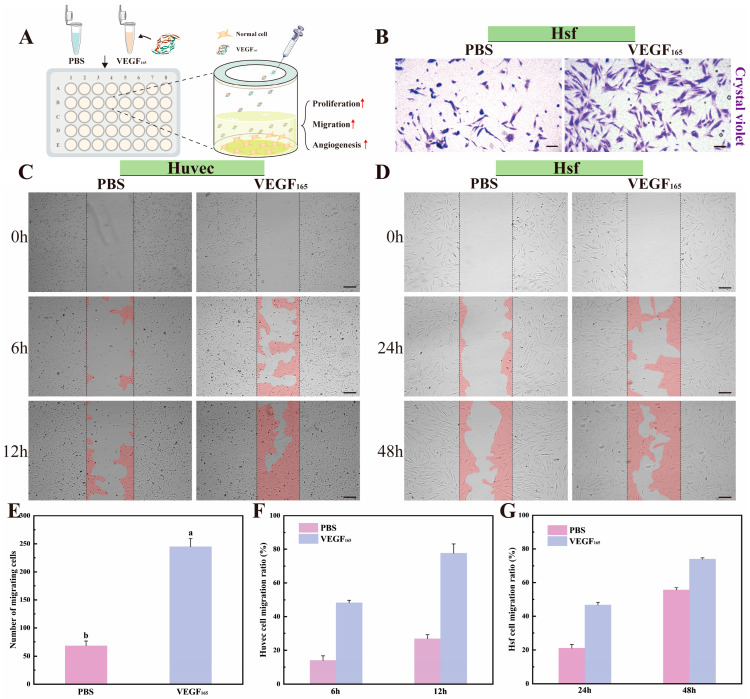
Functional validation of recombinant VEGF_165_ in promoting cell migration and wound healing. (**A**) Schematic workflow illustrating the experimental procedure: purified recombinant VEGF_165_ protein was added to cell culture media to assess its effects on proliferation, migration, and tube formation. (**B**) Representative images showing VEGF_165_-enhanced migration of HSF cells; scale bar, 200 µm. (**C**) Representative scratch wound assay images of HUVECs at indicated time points (0, 6, 12 h); scale bar, 200 µm. (**D**) Representative scratch wound assay images of HSF cells at indicated time points (0, 24, 48 h); scale bar, 200 µm. (**E**) Quantitative analysis of HSF cell migration. (**F**,**G**) Quantitative histograms of the scratch wound closure area for HUVEC (**F**) and HSF (**G**) cells over time. Data are presented as mean ± SD; statistical significance between groups is denoted by lowercase letters at *p* < 0.05 (*n* = 3).

In addition to its proliferative and pro-angiogenic effects, the capacity of cell-free synthesized VEGF_165_ to promote cell migration was evaluated using complementary assays, as outlined in the schematic workflow (Figure 7A). A Transwell migration assay demonstrated that VEGF_165_ treatment significantly increased the migration of HSFs, which are pivotal in tissue repair (Figure 7B). Quantitative analysis confirmed this robust pro-migratory effect (Figure 7E). This activity is likely mediated through VEGF_165_ binding to its cognate receptors, activating downstream pathways such as PI3K/Akt, which are instrumental in directing cell movement [45]. Furthermore, scratch wound healing assays were performed on both HUVECs and HSFs. VEGF_165_ markedly accelerated wound closure in HUVECs compared to the PBS control group, with near-complete coverage of the scratch area observed within 12 h (Figure 7C). A similar promotive trend was observed in HSFs, albeit over a different, cell-type-dependent timeframe (Figure 7D). Quantitative analysis of the scratch area over time provided conclusive evidence that VEGF_165_ significantly enhanced the migration rate of both cell types (Figure 7F,G). The pronounced response in HUVECs may be attributed to their high intrinsic migratory potential and abundant expression of VEGF receptors, enabling efficient chemotactic response [46]. These findings align with established literature wherein bioactive compounds promote motility by activating key signaling cascades such as PI3K/Akt and Rho/ROCK [47]. In summary, recombinant VEGF_165_ produced by the optimized wheat germ cell-free system exhibits potent biological activity, effectively promoting cell migration—a critical function for wound healing and tissue regeneration—thereby validating the platform’s capability to generate functional proteins.

## 4. Discussion

Here we present a metabolically engineered platform for efficient ATP regeneration based on a heterologous three-enzyme cascade- AK, ADK and ACK-expressed in *E. coli*. This whole-cell biocatalyst converts the low-cost precursors adenosine and acetyl phosphate into ATP with a yield of 76.31% following systematic parameter optimization. By directly mitigating ADP accumulation and energy depletion, the system reduces the conventional reliance on costly exogenous ATP in cell-free reactions. Coupling ATP-regenerating lysates from this biocatalyst with a wheat-germ CFPS system established a stable and economical energy-supply platform. Using this integrated approach, we produced the therapeutic protein VEGF_165_, confirmed its structural integrity and validated its biological activity—demonstrating the practical utility of the platform for synthesizing functional proteins in a cost-effective manner.

The construction of the ATP-regeneration module provided key insights into heterologous expression strategies in *E. coli*. Comparative analysis of two plasmid constructs, pET28a-aadck and pET28a-AADCK, revealed the critical role of translation-initiation control. In pET28a-aadck, AK was strongly expressed whereas ADK and ACK levels were extremely low-a disparity attributed to suboptimal RBS design near the start codon. The unusually short spacing between the Shine–Dalgarno (SD) sequence and the AUG likely hindered efficient assembly of the translation-initiation complex. This observation aligns with established principles that the SD–AUG distance and sequence context are key determinants of translation efficiency; appropriately increasing this spacing can substantially enhance heterologous protein yields [29,48,49]. Future efforts should therefore employ systematic screening or rational design (e.g., using RBS calculators) to optimize the RBS sequence and SD–AUG spacing for each enzyme, enabling balanced, high-level expression in *E. coli* [50].

By contrast, the pET28a-AADCK construct employed an IRES from encephalomyocarditis virus (EMCV) to drive translation independently of canonical prokaryotic signals, achieving robust expression of all three enzymes. Notably, certain eukaryotic IRES elements retain function in prokaryotes-for example, the IRES from *Plautiastali* intestine virus (PSIV) can initiate translation in *E. coli*, relying largely on its compact tertiary structure [51]. Subsequent studies demonstrated that this activity depends on ribosomal protein S1 and SD-like sequences upstream of the start codon [52]. Two regions within the PSIV IRES complementary to 16S rRNA are critical for its function in *E. coli*; similarly, IRES elements from *S. cerevisiae* (GPR1, NCE102, HAP4 and FLO8) also mediate prokaryotic translation, albeit with lower efficiency [53]. The EMCV IRES itself has been shown to support translation in shuttle plasmids [31]. Furthermore, the P2A peptide-widely used for co-translational “self-cleavage” in eukaryotic systems-produced an ADK-ACK fusion protein in the prokaryotic environment that retained full catalytic activity for ATP synthesis. This finding is consistent with previous reports of functional fusion proteins generated by 2A peptides in *E. coli*; for instance, expression of the *ectABC* gene cluster from *Nocardiopsis dassonvillei* NCIM 5124 using a P2A linker yielded a fusion of *EctC* and *EctA* that still enabled ectoine production at 131.41 mg/g CDW after IPTG induction [32]. Collectively, this comparative analysis underscores the importance of selecting appropriate expression elements and offers a practical guide for engineering complex multi-enzyme pathways in bacterial hosts.

Although *E. coli* is generally suboptimal for expressing complex eukaryotic proteins requiring extensive post-translational modifications, it remains a robust and widely used host for producing functionally active enzymes with simpler architectures. Numerous examples support this, including the successful recombinant expression in *E. coli* of proteins such as nicotinamide riboside kinase [54], adenosine kinase [55], nuclear export factors [56], NADH kinase [57], asparaginase II [58] and antibody recombinant fragments [59]-all originating from *S. Cerevisiae* or other eukaryotic sources while retaining high biological activity. Notably, soluble protein fraction correlates positively with enzymatic specific activity [60]. In our system, induction at 25 °C in *E. coli* carrying pET28-AADCK yielded a soluble enzyme fraction of up to 41.96% (Figure 2). Based on these observations, we adopted a whole-cell biotransformation strategy for ATP synthesis. A key finding during preliminary evaluation was that, although a portion of the target protein remained insoluble after induction at 25 °C, the enzymatic activity in the clarified lysate was sufficient for catalysis. When intact, induced cells were used directly, the ATP concentration produced (~7 mM) substantially exceeded the typical requirement (~1.2 mM) for conventional CFPS systems [61,62].

The whole-cell biocatalyst developed here is designed primarily for in vitro, cell-free biocatalytic applications, specifically as an energy-regeneration module for CFPS. In standard CFPS configurations, the core extract—whether derived from *E. coli* S30 or wheat germ—already contains a complex endogenous enzymatic network from the source organism. Previous studies have established hybrid CFPS platforms capable of producing eukaryotic proteins [63,64,65]. We therefore conceptualize our whole-cell catalyst as an external “energy-supply” module compatible with, for example, wheat-germ-based CFPS systems. Thus, at this proof-of-concept stage, we have focused on its core functional ATP supply-and its operational compatibility with CFPS reactions. Deeper investigations into the in vivo state, localization, stability and potential membrane interactions of these enzymes in the whole-cell context would represent valuable follow-up basic research.

Under normal physiological conditions, bacteria do not actively export ATP, as ATP serves as the universal cellular energy currency whose synthesis—primarily at the inner membrane or within the cytoplasm—and consumption are tightly regulated intracellular processes. Deliberate efflux would constitute an energy leak detrimental to cellular viability. However, high-level expression of heterologous proteins coupled with accelerated substrate metabolism can impose substantial stress on membrane integrity, leading to a nonspecific increase in permeability. This allows small molecules such as ATP to passively diffuse out of the cell-a phenomenon that is not a regulated physiological event but rather an incidental effect associated with high metabolic load or mild cellular stress. Similar metabolite “leakage” has been documented in metabolic engineering studies of *E. coli*, particularly during the overproduction of compounds including L-glutathione [66], naringenin [67], S-adenosyl methionine [68], leucine–leucine dipeptide [69], 2-O-α-d-glucosyl-sn-glycerol [60], xylonic acid [70], creatine [71], γ-aminobutyric acid [72] and L-theanine [73]. Furthermore, pretreatments such as thermal, freezing or surfactant exposure—commonly employed before whole-cell catalysis—can further alter membrane permeability and enhance extracellular accumulation of target compounds [17,72,74,75]. In the present study, cells were washed with a borax solution before catalytic use; even low concentrations of borax are known to disrupt bacterial membrane architecture and increase permeability [76]. Collectively, metabolic-burden-induced membrane perturbation and borax-mediated permeabilization provide a coherent explanation for the detection of ATP in the supernatant after whole-cell biotransformation.

Developing stable, efficient and economical energy-supply systems remains a central challenge in cell-free synthetic biology, especially for enabling more complex applications. Established energy-regeneration systems, such as the phosphocreatine/creatine kinase (Cr/CrK) couple, have long been the standard choice for platforms like WGE-based protein synthesis owing to their reliability. Exploring modular and engineerable energy-delivery strategies is, however, critical for building next-generation cell-free biomanufacturing platforms with greater flexibility and integration. The present study represents an initial step in this direction, focusing on constructing and functionally validating a modular “energy-generating unit” and exploring its potential synergy with classical regeneration systems.

This approach aligns with the emerging trend toward hybrid or modular cell-free configurations. Previous work has shown that combining extracts or enzyme systems from different origins can yield synergistic advantages-for example, mixing *E. coli* S30 with WGE to produce correctly folded eukaryotic proteins [64], supplementing yeast cell-free systems with bacterial lysates expressing specific enzymes to enhance pathway efficiency [65], or creating novel reaction systems from blended *E. coli* and cyanobacterial lysates [77]. Collectively, these studies illustrate a viable route for customizing cell-free systems through functional-module assembly. Following this paradigm, we engineered a configurable bacterial cell module designed for controllable ATP accumulation. Our results demonstrate that metabolically reprogrammed cells can achieve efficient ATP production in vitro, validating the core functionality of this energy-supply unit.

The ACK/AK cascade reaction employed in this study generates equimolar acetate as a byproduct during ATP regeneration. Acetate has been reported to inhibit cell-free translation systems, making it essential to evaluate its potential impact [1]. Importantly, in our experimental design, acetate production and the WGE translation reaction are temporally and spatially separated: acetate is generated primarily during the 3.5 h ATP production module, reaching a theoretical concentration of approximately 7.5 mM (Figure 3A). During the subsequent WGE translation reaction, energy regeneration relies exclusively on the Cr/CrK system, as adenosine and acetyl phosphate are not supplemented, thereby preventing further acetate generation. The acetate carried over into the translation reaction is diluted four- to six-fold to a final concentration of approximately 1.5–2.0 mM—substantially below levels of byproduct accumulation reported to inhibit protein production [78]. Furthermore, the WGE reaction system employs HEPES buffer (pH 7.8), which exhibits excellent buffering capacity in the pH 7.4–8.2 range. The system also contains 100 mM potassium acetate, indicating inherent tolerance of the translation machinery to acetate ions [9,27]. Under these conditions, we successfully expressed multiple functional proteins (Figure 4, Figure 6 and Figure 7), demonstrating that WGE-based in vitro translation was sustained for 15 h. In addition, borax in aqueous solution forms a boric acid/borate buffer system, and borate ions can form reversible ester complexes with molecules containing cis-diol groups (e.g., ribose, nucleotides, and RNA), potentially affecting nucleotide availability, mRNA secondary structure stability, ribosome recognition efficiency, and pH buffering capacity [79,80,81]. In the present study, borax was used only in the initial cell washing step. Following multiple processing steps—including centrifugation, resuspension in PBS, ATP production reaction, supernatant collection, and dilution—the residual borax concentration carried into the WGE translation reaction was negligible. It has been reported that formation of the 2:1 borate–ribose complex is favored at pH 12 with a high binding constant [80], and that high concentrations (500 μM) of boric acid induce global ribosome stalling at stop codons [82]—far exceeding any potential residual concentration in our system. In addition, a particularly instructive finding emerged during optimization of WGE-based reactions. As shown in Figure 5, pre-incubating reaction components with lysate from the engineered cells-which contains an enriched endogenous ATP/ADP pool-allowed the amounts of exogenous phosphocreatine and creatine kinase required to maintain comparable translation efficiency to be reduced to 25% and 50% of conventional levels, respectively. This observation indicates that an externally supplied, dynamic ATP/ADP pool can act synergistically with the endogenous Cr/CrK system, significantly lowering reliance on high concentrations of both the energy-storage substrate (phosphocreatine) and the regeneration enzyme (creatine kinase). Beyond offering a potential route to reduce material costs-especially relevant for larger-scale applications-this finding suggests that introducing an external energy-buffering or supply module could help reshape and optimize energy flux throughout the reaction network, thereby improving overall energy-utilization efficiency and robustness.

Indeed, although integrating the ATP-generating module substantially increased protein output relative to our baseline, unoptimized homemade WGE setup, the final GFP yield (~240 ng) remains considerably lower than that achieved by highly optimized commercial WGE systems (~1.6 mg) [9]. This clearly indicates that our first-generation system is not yet competitive in contexts where maximal protein production is the primary objective-a limitation likely arising from the added complexity of bacterial lysates and the still-unoptimized interplay between modules. For commercial systems, it is roughly estimated that GFP production reaches approximately 3 mg per mL of extract in a bilayer in vitro translation system. Commercial kits typically provide 1 mL of extract, and based on current pricing, the estimated cost per μg of GFP is approximately 9 RMB. In our optimized system, 240 ng of GFP was produced from 10 μL of wheat germ extract, corresponding to a yield of 24 μg per mL of extract. Considering the cost of all reagents from mRNA construction to in vitro translation, a 120 μL reaction costs approximately 12 RMB, which translates to an estimated cost of 5 RMB per μg of GFP—significantly lower than that of commercial kits. Nevertheless, the process of preparing WGE is labor-intensive and time-consuming, and it often exhibits inconsistent expression efficiency across different batches. The central aim of this work was not to rival the yield benchmarks of commercial platforms at this stage, but to demonstrate the feasibility of a modular integration strategy. We have successfully coupled a heterologously engineered energy-regeneration module with eukaryotic translation machinery and shown that it can effectively drive a previously underperforming homemade WGE system to achieve a significant increase in output (Figure 5). Future work will focus on refining this architecture-for instance, by employing encapsulation techniques to isolate potential side-effects from the bacterial module, or by rationally designing the metabolic interface between modules. The eventual promise of such a strategy may lie in specialized synthetic applications where extreme cost sensitivity, dynamic energy management or the coupling of complex pathways are more critical than raw yield.

We acknowledge that the current whole-cell strategy, which relies on phenotypic screening, requires improvement in robustness and generalizability. Future work will therefore focus on several complementary directions. First, at a fundamental level, protein-engineering and membrane-engineering approaches will be employed to enhance the intrinsic solubility and/or membrane- or surface-localization of key enzymes, thereby improving the specific activity and stability of the whole-cell catalyst. Second, at an applied level, the system will be integrated with diverse CFPS platforms and additional ATP-dependent biosynthetic pathways to systematically evaluate its performance as a versatile “energy cartridge”. In summary, this study provides a feasible route toward constructing an efficient, low-cost ATP supply system, clarifies its current practical and economic limitations, and outlines clear directions for future optimization.

## 5. Conclusions

This study establishes an integrated platform for biocatalytic ATP regeneration and subsequent cell-free synthesis of functional proteins. First, we engineered an *E. coli* whole-cell biocatalyst expressing a heterologous three-enzyme cascade (AK, ADK, ACK), optimizing its catalytic parameters to efficiently produce ATP. This ATP-enriched reaction lysate then served as the foundational energy source for a refined WGE-based CFPS system. This optimized CFPS platform successfully produced multiple recombinant proteins, with VEGF_165_ serving as a primary functional model. The cell-free synthesized VEGF_165_ was not only non-cytotoxic but also exhibited potent bioactivity. It significantly promoted the proliferation of HSFs and HUVECs. Crucially, it demonstrated a robust pro-angiogenic effect, dramatically enhancing the formation of capillary-like structures by HUVECs in vitro, as quantified by increases in total tube length and branch point number. Furthermore, VEGF_165_ effectively accelerated the migration of both HUVECs and HSFs in wound-healing assays, a key cellular process for tissue repair. Collectively, these findings validate the efficacy of the sequential ATP regeneration and CFPS system, demonstrating its capability to produce biologically active, therapeutic proteins with significant potential for applications in wound healing and regenerative medicine.

## Figures and Tables

**Table 1:** Comparison of different CFPS systems.

System	Advantages	Disadvantages	Suppliers	Yield Range
*E. coli*	1. Well-established 2. Simple cultivation and fast cell growth and lysate preparation 3. Low cost 4. Easy genetic engineering 5. Well-established 6. high protein yield 7. Fast translation speed	1. No post-translational modifications 2. No endogenous membrane structures for the synthesis of integral membrane proteins 3. Eukaryotic proteins may be insoluble 4. codons need to be optimized	RTS (5 PRIME); Expressway (Life Technologies, Carlsbad, CA, USA); S30 T7 high yield (Promega, Madison, WI, USA)	100 μg/mL to 1 mg/mL
Pure	1. High purity 2. Low background interference 3. High scalability	1. High cost 2. No post-translational modifications 3. Lack of molecular chaperones and auxiliary folding	PURExpress (New England Biolabs, Ipswich, MA, USA); PURESYSTEM (BioComber, Tokyo, Japan)	50–200 μg/mL
Human cell	1. Correct folding and assembly of human proteins 2. Containing endogenous microsomes 3. Similar post-translational modifications in human	1. Low protein production 2. High cost 3. Complex cultivation techniques	One-step human IVT (Thermo Scientific, Waltham, MA, USA)	Up to 750 μg/mL
Wheat germ	1. High protein yield 2. Production of complex proteins 3. High protein solubility 4. Cap independent translation 5. Well-established 6. Often, no codon optimization is needed 7. High scalability	1. Time-consuming preparation of the extract 2. Limited post-translational modifications 3. Absence of endogenous membrane structure	WEPRO (CellFree Sciences, Yokohama, Japan); TNT-coupled (Promega); RTS CECF (5 PRIME)	Up to 10 mg/mL
Rabbit reticulocyte	1. Well-established 2. Cap independent translation 3. Mammalian system	1. Low protein yield 2. Post-protein modification requires supplementation of microsomes 3. Sensitive to additives 4. No glycosylation 5. High hemoglobin concentration 6. Extract requires live animals	TNT Coupled (Promega); Retic lysate IVT (Life Technologies, Carlsbad, CA, USA)	<100 μg/mL
Insect cell	1. Post-translational modification of proteins 2. Cap independent translation 3. Disulfide bond modification 4. Endogenous microsomes are available 5. Synthesis of membrane proteins	1. Low protein production 2. High cost 3. New system	TNT T7 (Promega); EasyXpress Insect kit (Qiagen, Beijing, China/RiNA)	<100 μg/mL

**Table 2:** List of strains, plasmids and oligonucleotide primers.

Type	Identity	Relevant Characteristics
Strain	*E. coli* DH5α	F-φ80 lac ZΔM15 Δ(lacZYA-arg F) U169 endA1 recA1 hsdR17(rk-,mk+) supE44λ-thi-1 gyrA96 relA1 phoA
	*E. coli* BL (DE3)	F-ompT hsdSB(rB-mB-) gal dcm(DE3)
Plasmid	pET28a	Expression vector, KanR
	pEU-E01	Expression vector, AmpR
	pET28a-aadck	pET28a plasmid carrying AK, RBS, ADK, RBS, and ACK genes, KanR
	pET28a-AADCK	pET28a plasmid carrying AK, IRES, ADK, P2A, and ACK genes, KanR
	pEU-VHb	pEU-E01 plasmid carrying full-length VHb gene, AmpR
	pEU-LegH	pEU-E01 plasmid carrying full-length LegH gene, AmpR
	pEU-sfGFP	pEU-E01 plasmid carrying full-length sfGFP gene, AmpR
	pEU-PH	pEU-E01 plasmid carrying full-length PH gene, AmpR
	pEU-BDNF	pEU-E01 plasmid carrying full-length BDNF gene, AmpR
	pEU-mCherry	pEU-E01 plasmid carrying full-length mCherry gene, AmpR
	pEU-VEGF_165_	pEU-E01 plasmid carrying full-length VEGF_165_ gene, AmpR
Primer	f-ak	CGCGGATCC**ATG**ACCGCGCCGCTGGTT
	r-ak	TAAAGTTAAACAAATTA*ATGGTGGTGGTGGTGGTG*TTTAGAATAGCTAATT
	f-adk	ATTTGTTTAACTTTAAGAAGGAGA**ATG**AGCTCTTCTGAATCTATTCGTATG
	r-adk	TTAAAGTTAAACAAATTA*GTGGTGATGGTGGTGGTG*GTCTTTACCCAGTTTG
	f-ack	TTTGTTTAACTTTAAGAAGGAGA**ATG**TCCTCCAAACTGGTTCTGGTTCT
	r-ack	GTGCTCGAGTTA*GTGGTGGTGGTGATGGTG*GGCGGTCAGACGGCTAGC
	F-AK	CGCGGATCC**ATG**ACCGCACCATTGGTAGTATTGGGTA
	R-AK	GGGGAGGGAGAGGGGCTA*GTGATGATGATGATGATG*TTTAGAGTAAGATAT
	F-IR	CCCCTCTCCCTCCCCCCCCCCTAACGTTA
	R-IR	TGGATTCTGAGCTAGACATATTATCATCGTGTTTTTCAAAGGA
	F-ADK	**ATG**TCTAGCTCAGAATCCATTAGAATGGTCCTAATTGG
	R-ADK	TCAGCAGAGAGAAGTTAGTCGCACCAGAACCAGA*GTGATGATGATGATGATG*ATCCT
	F-ACK	TCTCTCTGCTGAAGCAGGCTGGTGACGTTGAAGAAAACCCAGGTCCG**ATG**TCGAGTAAGT
	R-ACK	TCTCACTCGAGTCA*GTGATGATGATGATGATG*GGCAGTCAGGCGGCTCG
	F-BCP	TCCATGACCGCACCATTGGTAGTATTGGGTAACC
	R-BCP	CTGTGAGCGGCGCCTGCACCTAAAGCC
	F-VHb	CGATATCTCGAGA**ATG**TTAGACCAGCAAACCATTAACA
	R-VHb	CCCGGGATCCTTA*GTGGTGGTGGTGGTGGTG*TTCAACCGCTTGAG
	F-LegH	ATATCTCGAGA**ATG**GGCGCCTTCACTGAAAAAC
	R-LegH	CCGGGATCCTTA*GTGGTGGTGGTGGTGGTG*GAACGCTTTTTTGATA
	F-GFP	ATATCTCGAGA**ATG**CGTAAAGGCGAAGAACTGTTTAC
	R-GFP	CCGGGATCCCTA*GTGGTGGTGGTGGTGGTG*AGCCACCAGTGCGTA
	F-PH	TATCTCGAGA**ATG**GTGCATCTGTCTGCTGAGGAGAA
	R-PH	CGCGGTACCTTA*ATGATGATGATGATGGTG*GTACTTGTGGGCCA
	F-BDNF	TATCTCGAGA**ATG**CACAGCGACCCAGCTAGAAGAGG
	R-BDN	CCGGGATCCTCA*GTGGTGGTGGTGGTGGTG*CCGGCCTCTCTTGA
	F-mCh	CGAGGATCC**ATG**GTGAGCAAGGGCGAGGAGGAT
	R-mCh	CTAGTGCGGCCGCTTA*GTGATGATGATGATGATG*CTTGTACAGCTCGTCCA
	F-165	ATATCTCGAG**ATG**GCTCCGATGGCAGAAGGCG
	R-165	TAGTGCGGCCGCTTA*GTGATGATGATGATGATG*ACGGCGTGGTTTG
	F-Trans	GCGTAGCATTTAGGTGACACTATAGAACTCAC
	R-Trans	TTTTTTTTTTTTTTTTTTTTTTTTTTTTTTGCTCGATCGA

Note: AmpR represents ampicillin resistance; KanR denotes kanamycin resistance; underlined sequences represent restriction enzyme sites; bold black text represents start codons; italicised text represents 6 × His tags.

## Data Availability

The original contributions presented in this study are included in the article. Further inquiries can be directed to the corresponding author.
